# The lungs need to be deflated: effects of glycopyrronium on lung hyperinflation in COPD patients

**DOI:** 10.1186/2049-6958-9-19

**Published:** 2014-04-01

**Authors:** Claudio M Sanguinetti

**Affiliations:** 1 Consultant Respiratory Medicine, Quisisana Clinical Center, Via G. Porro 5, Rome 00197, Italy; 2 Previously Director, Pneumology and Respiratory Intensive Care Unit, San Filippo Neri General Hospital, Rome, Italy

**Keywords:** Bronchodilators, COPD, Dyspnea, Glycopyrronium bromide, Inspiratory capacity, Lung hyperinflation

## Abstract

Chronic obstructive pulmonary disease (COPD) is characterized by persistent airflow limitation caused by bronchial alterations, small airways disease and parenchymal destruction. In patients with COPD the structural and functional lung alterations can progress more or less rapidly from the initial small airways disease to an overt COPD where a severe expiratory flow limitation takes place. In these conditions, lung hyperinflation develops characterized by increase in functional residual capacity (FRC) and decrease in inspiratory capacity (IC). Thus, IC is an easy and reliable index to monitor lung hyperinflation and to assess the efficacy of bronchodilator drugs. When FRC increases, tidal volume (VT) is located in a more flatted upper part of the P –V curve of the respiratory system and respiratory muscles must sustain a greater elastic workload. Furthermore, due to inadequate time for expiration, there is a positive alveolar pressure at the end of expiration (PEEPi). This represents a further elastic workload for the inspiratory muscles. This impairment of ventilatory mechanics generates dyspnea that in most severely compromised patients occurs also for small efforts causing activity limitation and worst health-related quality of life (HRQoL). Due to these respiratory alterations, bronchodilators are the cornerstone of the long-term treatment of COPD in order to decrease airways resistances, lung hyperinflation and exacerbation rate, and improve patient’s symptoms, exercise tolerance and health status. Long-acting antimuscarinic bronchodilators (LAMAs) have proven to be very useful in terms of lung deflation and exercise tolerance. Recently, new LAMAs with several positive characteristics have been introduced into clinical use among which glycopyrronium bromide has shown to be particularly effective. Glycopyrronium has a longer-lasting effect compared to other anticholinergic drugs, therefore it allows a single daily administration and facilitates the therapy of a disease that needs a chronic bronchodilation by decreasing the mechanic stress of the airways determined by repeated bronchoconstriction and increasing patient’s adherence to treatment plan with better clinical results. Several studies demonstrated that glycopyrronium is able to positively and significantly decrease lung hyperinflation, symptoms, and improve psycho-physical status of COPD patients, with a low rate of adverse events, similar to that of placebo.

## Review

Chronic obstructive pulmonary disease (COPD) is a pathological respiratory condition characterized by persistent airflow limitation caused in various measures by bronchial alterations (chronic bronchitis), small airways disease and parenchymal destruction (pulmonary emphysema). The disease is determined by a chronic abnormal response to noxious inhaled substances, mainly tobacco smoke, presents with persistent cough, sputum production, dyspnea and decreased exercise tolerance, and is associated with various complications and comorbidities, especially cardiovascular and metabolic [1-3]. COPD charges a relevant social and economic burden, affecting almost 4.5% of population in Italy [1]. The main symptom of COPD is dyspnea and the patients reduce their daily activities in an attempt to relieve this symptom; but by avoiding the physical activity patient enters a vicious circle which leads to deconditioning and having more dyspnea.

This article addresses the respiratory alterations occurring in COPD that lead to lung hyperinflation and dyspnea, their pathophysiologic and clinical consequences and the role of bronchodilators with a particular focus on glycopyrronium at improving health status and health related quality of life of COPD patients by decreasing the hyperinflation.

### Pathophysiology and consequences of lung hyperinflation in COPD

**Figure 1:**
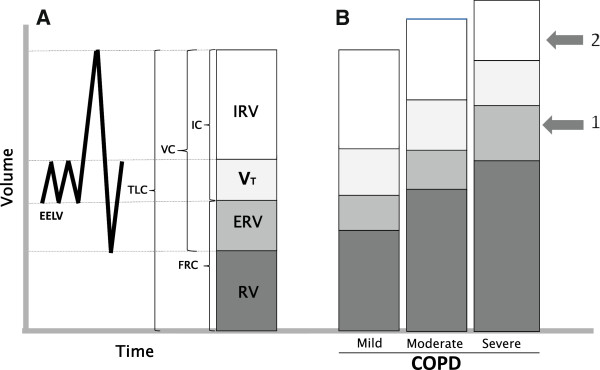
**Lung volumes and capacities in normal subjects and in COPD patients. A)** Lung volumes and capacities in healthy subject: EELV, end-expiratory lung volume, ERV, expiratory reserve volume; FRC, functional residual capacity; IC, inspiratory capacity; IRV, inspiratory reserve volume; RV, residual volume; TLC, total lung capacity; VC, vital capacity; VT, tidal volume. **B)** Progressive increasing in static lung hyperinflation in relation to severity of COPD; arrow 1 indicates the threshold of dyspnea onset; arrow 2 indicates the limit of dyspnea tolerance. Modified from [19].

The volume of air introduced into the lungs during quiet breathing (tidal volume, VT) is sufficient for pulmonary ventilation and gas exchange. The amount of air present in the lungs at the end of a normal expiration is the functional residual capacity (FRC) that includes expiratory reserve volume (ERV) and residual volume (RV, the volume of air remaining in the lungs after a deep expiration), which is a result of the force displayed by expiratory muscles in the healthy young people to overcome chest wall elasticity, while in the elderly it increases due to a reduced elastic force of the lung [4]. At this level, there is a static equilibrium between the lung and the chest cage, because the outwards force of chest cage is completely counterbalanced by the inwards force of pulmonary elastic recoil and this is defined as relaxation volume, i.e. the pulmonary pressure equals zero, and there is no flow through the airways. During exercise or in other situations when requested, the volume of inspiration increases also utilizing the inspiratory reserve (IRV) until the total lung capacity (TLC) is reached. TLC and VC (vital capacity, that is the maximum volume of air that is possible to mobilize with a deep inspiration followed by a deep expiration) are important reference indices in diseases causing a restrictive defect, as pulmonary fibrosis, respiratory muscle disorders, and chest wall alterations (Figure 1A).

In the volumetric partition of VC [5] particular value is now attributed to inspiratory capacity (IC, sum of VT and IRV) because, when reduced, it may testify a condition of lung hyperinflation (LH), caused by the increase in RV, FRC, and TLC [6], as frequently observed in COPD patients [7]. In these patients, IC showed a more significant correlation with the exercise tolerance than the forced expiratory volume in one second (FEV_1_) [8]. In addition, several studies demonstrated that IC, also when standardized for TLC (IC/TLC), as marker of lung hyperinflation both in resting conditions (static LH) and during exercise (dynamic LH),is an independent predictive factor of mortality in COPD patients [9,10], and also indicative of a longer hospital stay following thoracic surgery [11]. Therefore, an ideal bronchodilator should demonstrate to be able to decrease the hyperinflation of the patient, increase inspiratory capacity and, consequently, increase exercise tolerance. Based on many functional and clinical observations, IC, besides FEV_1_, is increasingly used as an index to assess the efficacy of bronchodilator drugs in COPD patients.

Several previous studies [12,13] demonstrated undeniably that the damage caused by the inhalation of toxic compounds, like cigarette smoke and environmental pollutants, primarily involves the “small airways”. This definition refers to bronchioles with an internal diameter equal to or lower than two millimeters, that is the terminal and respiratory bronchioles, thus a very peripheral site in the lung and very close to gas exchanging zone of pulmonary alveoli [14]. Patency of small airways is normally maintained, especially in expiration, by the integrity of bronchial walls that, while lacking in cartilagineous framework, collapse only when the lung empting is almost complete, and by the alveolar-airways attachments acting as elastic bands to maintain the bronchial calibre. In smokers, the structural and functional lung alterations progress more or less rapidly from the initial small airways disease to an overt COPD [15,16]. At the beginning of the disease, small airways closure may occasionally occur during tidal expiration, while with the progression of the disease this alteration is constant and associated with a severe expiratory flow limitation (EFL) [17,18]. EFL is caused by the increase in airways resistance consequence of a reduction of bronchial-bronchiolar caliber due to structural remodeling and augmented vagal tone, together with the destruction of elastic pulmonary tissue. The flows normally utilized are thus maximal,that is the maximum expiratory flow is within the tidal volume [18], and any further increase in pleural pressure does not increase the expiratory flow which is only dependent on the elastic recoil of the lung [19]. Since the elastic recoil pressure raises in parallel with the increase in lung volume,when EFL occurs patients must breath at a higher pulmonary volume to exploit the only mechanism able to increase their expiratory flow. In these conditions, residual volume increases due to the closure of the airways at higher pulmonary volume and consequently FRC increases because the volume at which the balance between the elastic pressures of the lung and chest wall occurs is increased, leading, starting from dynamic hyperinflation, eventually to a static lung hyperinflation (sLH). This has important implications in that the work of inspiratory muscles increases to counteract the augmented elasticity of lung tissue. In addition, the increased pulmonary volume determines a shortening of the inspiratory muscles which consequently generate a lower pressure for a certain stimulus. In the natural history of the anatomic and functional damage of COPD a progressive alteration of pulmonary volumes occurs characterized by a progressive increase in FRC and parallel decrease in IC until the patient inevitably develops dyspnea even during quiet breathing and it is impossible to increase the extent of ventilation beyond a certain limit [20] (Figure 1B). Thus, lung hyperinflation and the consequent alterations of respiratory mechanics determine an increased respiratory work that in turn leads to fatigue of respiratory muscles that must sustain a greater load, with inefficiency of respiration and onset of respiratory failure, initially characterized only by hypoxemia and then also by hypercapnia. Dyspnea usually arises when gas exchange is inefficient as in ventilation/perfusion mismatching, exercise-induced hypoxemia, and impaired respiratory mechanics, where an uncoupling occurs between the increased ventilatory stimulus and the decreased mechanical performance. In the most severely compromised COPD patients, dyspnea occurs also for small efforts and consequently an activity limitation develops that leads to deconditioning and worsening of health-related quality of life (HRQoL) [20].

**Figure 2:**
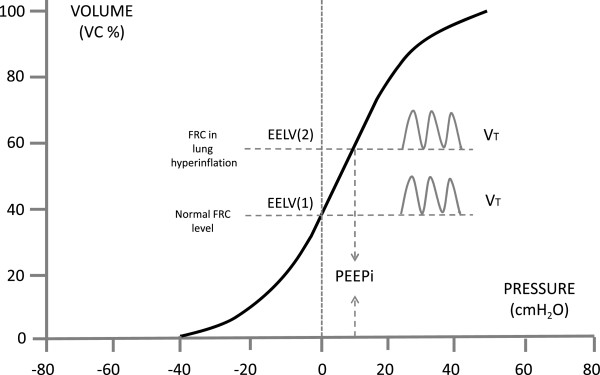
**Pressure-volume curve of the respiratory system.** EELV, end-expiratory lung volume: (1) in healthy subjects, (2) in subjects with lung hyperinflation; PEEPi, intrinsic end-expiratory positive pressure; VT, tidal volume.

To accomplish a normal expiration and reach the relaxation volume, the patient affected by EFL needs longer expiratory time as the expiratory flow is lower. Therefore, the inspiration starts at an end-expiratory lung volume (EELV) greater than the relaxation volume leading to dynamic lung hyperinflation (dLH) [21]. Thus, when EFL arises in COPD patients [22], they breath with progressively increased lung volumes (increased FRC and equally decreased IC). In this situation the activation of expiratory muscles, while not increasing the expiratory flow, may aggravate the dyspnea perception by collapsing intrathoracic airways beyond the bronchial closing point [23]. When EELV increases, normal breathing takes place at a higher absolute lung volume and VT is situated in a more flatted upper part of the pressure – volume curve of the respiratory system, such as for its attainment inspiratory muscles must sustain a greater elastic work. Furthermore, due to inadequate time for expiration, the relaxation volume is not reached and the mean alveolar pressure at the end of expiration exceeds the atmospheric pressure and has a positive value that is called intrinsic positive end-expiratory pressure (PEEPi). This represents a further elastic respiratory load for the inspiratory muscles. In fact, when inspiration begins inspiratory muscles must counterbalance this pressure load before generating a negative alveolar pressure that determines the inspiration and the achievement of VT. Thus, in COPD patients with dLH, while inspiratory muscles are able to generate a lower pressure for a given stimulus due to their anatomic and functional change caused by LH, they have to sustain a greater workload in relation to the increased pulmonary volume at which VT is fulfilled and to the threshold load charged by PEEPi [17,18,24,25] (Figure 2). The level of PEEPi has been found to be correlated with the resting hypercapnia [26].

When these dynamic conditions develop, a rapid increase in FRC takes place caused by air trapping at the end of expiration and dynamic hyperinflation of the lungs, together with a parallel decrease in IC, because patients increase the breathing frequency and further shorten the expiratory time. In fact, while in the normal subject at the onset of an exercise there is a fall of EELV, such as the respiratory system remains on the steeper part of the pressure-volume relationship, and VT increases also utilizing part of the inspiratory reserve (IRV), in a subject with EFL and LH tidal volume cannot be increased beyond a certain level because any further elevation of pressure cannot generate any volume increase [18,27]. However, the breathing frequency is augmented as a compensation attempt, resulting in further rise of EELV. In addition, the new positioning of VT along the pressure-volume curve of the respiratory system is such that VT lies closer to TLC, and the IRV is decreased. This is a further limiting factor because the “dyspnea limit”, that is the volume level at which dyspnea becomes unbearable, has been shown to be when IRV is lower than half a litre [28]. Bronchodilators, the cardinal of COPD therapy, break this vicious circle by reducing the airway obstruction, which leads to decreasing of the residual volume allowing patients longer exercise time which has many beneficial aspects for the patients daily life and disease progress. A significant correlation also appeared between the IC decrease and the dyspnea presence and degree both during exercise testing and during normal daily activities in COPD patients [29].

### Effects of glycopyrronium on lung hyperinflation and its consequences

Due to persistent airflow limitation in COPD, bronchodilators, especially the long-acting ones, are the cornerstone of the long-term treatment of this disease, with the aim of decreasing to the minimum the airways resistances and improving the parameters closely correlated with the patient’s health status and prognosis, like symptoms, acute exacerbations, exercise tolerance and physical and psychic general conditions.

The efficacy of long-acting bronchodilators in COPD has been extensively documented in studies performed both with beta2-agonists (LABAs) [30,31] and with muscarinic antagonists (LAMAs) [32,33], as the cholinergic tone is recognized as the major reversible component of the airflow obstruction in this disease [34]. In almost all studies the primary outcome measure to assess the drug efficacy was the FEV_1_, whose changes however are poorly correlated with the variations of symptoms and exercise tolerance, which instead are related to changes of lung hyperinflation [35,36]. Therefore, in order to assess the efficacy of a bronchodilator in COPD, the physiologically more reliable parameter is the inspiratory capacity, which correlates inversely with FRC and can thus be considered a marker of changes of LH.

In fact, it has been demonstrated that LH is, at least in part, reversible with administration of bronchodilators, and lung deflation causes an increase in IC and symptoms improvement in COPD patients. In these patients, the increase in FEV_1_ after bronchodilation is generally small, if any, while the most important effect is the increase in IC, which sustains the symptoms improvement, even if the indices expressing the rate of bronchial caliber, like the FEV_1_/FVC ratio, may sometimes be scarcely improved [37,38]. Noteworthy is the observation that FRC increases exponentially with the progressive reduction of FEV_1_ and the most significant change after bronchodilation is the decrease in FRC and RV, that is in LH, independently from the basal FEV_1_ value [27]. During dynamic conditions it has been also observed that the prolongation of “endurance time” (ET), *i.e.* the span of time in which the exercise is tolerated, is more related to the effect of bronchodilators than other parameters assessed during cycloergometer exercise or 6 minutes walking test (6MWT) [39]. This means that bronchodilators decrease directly the hyperinflation and increase the exercise capacity.

Beneficial effects on IC increase have been obtained also with beta2-agonists characterized by a particularly long duration (lasting 24 hours) of action (“ultra-LABA”) that allows a single daily administration. In a trial [40] comparing formoterol and indacaterol the latter at 300 mcg OD provided a greater effect on bronchial obstruction and LH than the former at usual dose of 12 mcg BID in patients with COPD. A short-term trial by Rossi et al. [41] eventually confirmed that indacaterol 150 mcg OD is capable of increasing IC significantly more than placebo. In the same study the effect was also numerically higher than tiotropium bromide (TB) without reaching significance. Interestingly, taking into account the maximum increase of IC, more patients with indacaterol exceeded 20% and 30% improvement compared to TB indicating that indacaterol is capable of producing great improvements in some patients.

On the other hand, also antimuscarinic bronchodilators have proven to be very useful in terms of lung deflation and exercise tolerance. Recently, new long-acting LAMAs with several positive characteristics have been introduced into clinical use among which glycopyrronium has shown to be particularly effective.

*Glycopyrronium bromide* (GB) has a quaternary ammonium structure and low oral bioavailability, that reduces the drug’s systemic effects [42]. Glycopyrronium is delivered by a dry-powder inhaler (DPI), the Breezhaler®, that has a low resistance and requests a lower inspiratory flow, thus easy to be utilized by COPD patients of different age and severity, and already widely used to inhale indacaterol dry-powder [43,44].

The longer-lasting effect of GB compared to other anticholinergic drugs allows a single daily administration, which can facilitate the therapy of a disease that needs a chronic bronchodilation by decreasing the mechanic stress of the airways avoiding repeated bronchoconstriction, and by increasing patient’s adherence to treatment plan and thus obtaining better clinical results [45-47].

**Figure 3:**
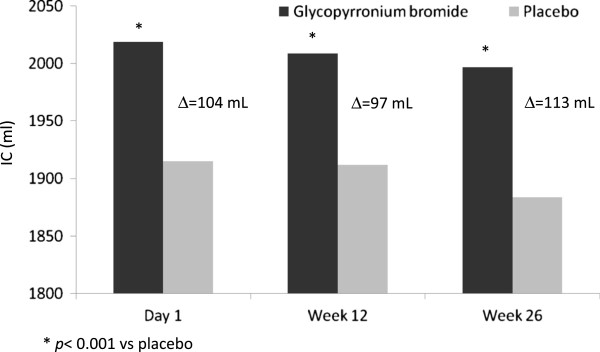
**Difference of inspiratory capacity (IC) values between glycopyrronium bromide and placebo at the end of the first day and at 12**^**th **^**and 26**^**th**^**week of therapy before the administration of the active drug or placebo.** Δ = Difference between glycopyrronium bromide and placebo.

In a first phase III randomized study [48] of 26 weeks with 822 patients with moderate-to-severe COPD, effectiveness, safety and tolerability of GB versus placebo were assessed. The study demonstrated that glycopyrronium rapidly and significantly increased trough FEV_1_ compared to placebo on the first day of therapy and remained elevated for 26 weeks. Mean trough FEV_1_ at the 12^th^ week, calculated as mean of values recorded between 23.15 and 23.45 hours, was significantly (p < 0.001) higher in patients given GB than in those treated with placebo, and the difference was 108 ± 14.8 mL, thus greater than the minimum clinically important difference (MCID) for FEV_1_ that is 100 mL [49], reaching 113 ± 16.5 at 26th week. Inspiratory capacity, an important indicator for hyperinflation, was also significantly improved by glycopyrronium reaching a difference of 104 mL versus placebo already at day 1 and maintaining this difference over 26 weeks (p < 0.001 vs. placebo in all cases) [48] (Figure 3), demonstrating that glycopyrronium is an effective bronchodilator which provides sustainable bronchodilation and decreases the hyperinflation. As a consequence of bronchodilation and LH decrease, at the 26^th^ week the dyspnea degree, measured as TDI (Transitional Dyspnea Index) score was significantly (p < 0.001) improved in COPD patients treated with glycopyrronium compared to those given placebo**,** exceeding even the limit of clinically important difference and suggesting that patients perceived the improvement. In addition, the rate of patients treated with GB presenting a clinically significant improvement of HRQoL measured with the SGRQ (Saint George’s Respiratory Questionnaire) was greater than that of patients who took placebo. As already underlined, lung hyperinflation causes dyspnea and reduces exercise tolerance in COPD patients and the increase in IC after GB administration is the evidence for the reduction of lung hyperinflation. The results of this study [48] indicate that glycopyrronium is capable of positively and significantly affecting lung hyperinflation, symptoms, and psycho-physical status of COPD patients, likely allowing them to better use the tidal volume and improve the respiratory performance, in presence of a rate of adverse events (AEs) lower than with placebo.

**Figure 4:**
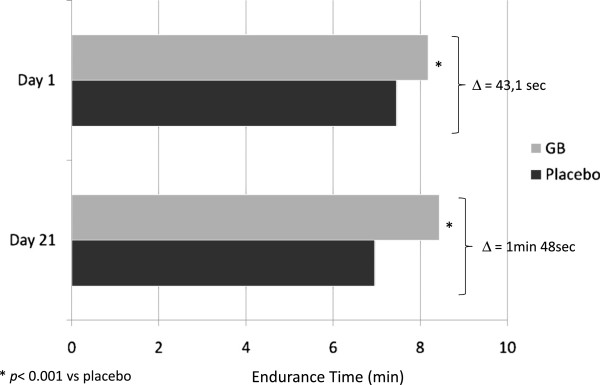
**Endurance time (ET) on the first and 21**^**st **^**day of therapy with glycopyrronium bromide (GB) or placebo in patients with moderate-to-severe COPD.** From the data of [52].

**Figure 5:**
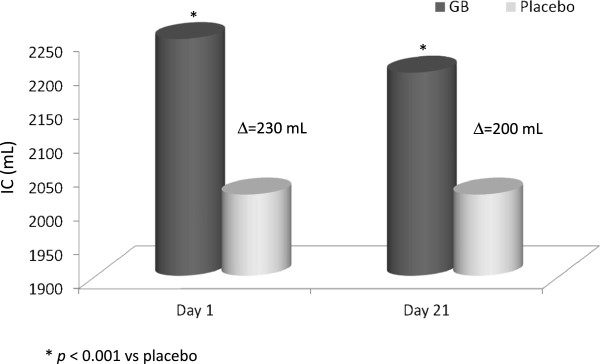
**Values of inspiratory capacity (IC) “isotime” during exercise on the first and 21**^**th **^**day of therapy with glycopyrronium bromide (GB) or placebo.** Modified from [53].

The favorable results obtained in this study represented the basis for a second trial [50] whose objective was to evaluate the efficacy and tolerability of GB, not only versus placebo but also in relation to TB given “*open-label”* for a period of 52 weeks in 1,066 patients with moderate to severe COPD. GB induced a very fast bronchodilation within 5 minutes after the first administration at the onset of therapy, greater than that caused by tiotropium and placebo (p < 0.01). At the 26^th^ week, bronchodilation induced by GB, measured as area under the curve (AUC) from the administration of the drug until the 4^th^ hour, was greater than that with TB and placebo (p < 0.01). Importantly, at the end of first day of therapy, and at 26^th^ and 52^nd^ week, the difference between GB and placebo was 91,134, and 108 mL respectively (p < 0.001), approaching rapidly the MCID and maintaining it over the time, whereas the significant (p < 0.001) difference between tiotropium and placebo was under the MCID over time (83, 84, and 89 mL respectively). The increase in IC with GB was greater than with placebo (p < 0.001) and similar to that with TB. Thus, the results of this second study demonstrated that the administration of glycopyrronium was effective on all set end-points and similar to that of TB, which at that moment was the only once daily LAMA available for long-term treatment of COPD with inhalant antimuscarinic drugs. However, GB differed in a greater rapidity of action and higher bronchodilating effects compared with TB on the first day of administration, which was maintained at the 12^th^, 26^th^, and 52^nd^ week. The faster bronchodilation after the administration of the drug may give the patient the possibility to carry out the morning activities easier, a period of the day which is considered the most worrisome by the majority of patients [51]. The benefit from the fast bronchodilating effect of GB, which maintains throughout the time and produces a decrease in lung hyperinflation and dyspnea, is not only particularly relevant for patients daily activities, previously limited by LH and its consequences, but it also favours a greater adherence to treatment.The consequences of reduced hyperinflation have been investigated in another multicentric, cross-over, and randomized phase III study of 21 days [52], whose main objective was to assess the effect of GB 50 mcg OD on exercise tolerance in patients with moderate-to-severe COPD, where the exercise tolerance and inspiratory capacity were measured as ET during a constant submaximal exercise test (SMET). Even on the first day, glycopyrronium allowed patients to tolerate exercise for a longer time than placebo (the difference was 43.1 seconds more with GB p < 0.001) and the time difference at the 21^st^ day was further increased (1 min and 29 sec more with GB, p < 0.001) (Figure 4). The greater exercise capacity observed in this study with GB administration, even in terms of lower muscular exhaustion during exertion, is really important also because, in relation to what emerged from other studies [53], patients can certainly perceive the improvement and become more active and less detached from the social framework, with improved quality of life. The increase in exercise tolerance is mainly caused by the effective and sustained bronchodilation afforded by GB (p < 0.05 vs. placebo) which on turn determines a decrease in airways resistance and lung hyperinflation, in this study witnessed by the significant increase in airways conductance and inspiratory capacity, calculated “*isotime”* during the exertion in the course both of GB and of placebo therapy. In fact, the inspiratory capacity during GB therapy increased more than 200 mL compared to placebo both at the beginning of the study and after 3 weeks (Figure 5) (52). This functional benefit turned into a symptoms improvement, with a significant and clinically important [54] decrease in dyspnea measured with Borg CR10 and TDI scores during treatment with glycopyrronium compared to placebo. An increase in IC of 200 mL or more is a remarkable benefit in patients with seriously limited respiratory operational volumes, because the elastic charge**,** and consequently the effort, is lower during the respiratory movement (LH decreases and respiratory reserve is better utilized) and dyspnea level decreases during normal daily activities. In addition, the incidence of adverse events (AEs) was similar to that of patients receiving placebo and the majority of AEs were mild or moderately severe and no death occurred during the study [52], thus confirming the safety of glycopyrronium as already demonstrated in previous studies where also serious AEs (SAEs) occurred with a lower frequency in the GB group compared with TB and placebo groups [50].

Quite recently, the results of this study have been confirmed by a new evaluation of efficacy and safety of glycopyrronium versus blinded tiotropium [55] in a 12-week study with 657 patients with moderate-to-severe COPD. The choice of blinding TB has been taken to minimize possible sources of bias that could arise in open-label studies. In fact, patients who know they are given an active drug or have had previous experience of it may be more prone to report favorable results, or may be influenced on their decision about remaining on treatment [56]. This was the first trial where GB has been compared with blinded TB. Briefly, following the first dose on the 1^st^ day of treatment GB produced greater FEV_1_ values than TB with least squares mean (LSM) differences of 51 mL and 63 mL compared to TB at 5 min and 15 min post-dose respectively (both p < 0.001), and FEV_1_ was greater with GB than with TB at all time points from 0 to 4 hours post-dose (p < 0.001). Glycopyrronium also determined a significantly higher increase in inspiratory capacity than tiotropium at 30 min (p < 0.001) and 2 hours (p < 0.001) after the dose administration indicating to a higher reduction of hyperinflation. At week 12 FEV_1_ and other spirometric variables were comparable between GB and TB, as well as TDI focal score, SGR total score, incidence of moderate or severe COPD exacerbations, whereas the mean daily total symptom score was significantly (p = 0.035) lower with GB than with TB. The safety of glycopyrronium was confirmed also in this recent investigation, because the overall incidence of AEs, SAEs and AEs leading to discontinuation was low and similar between the two treatment groups. This study designed to minimize the possible bias once more demonstrates that in patients with moderate-to-severe COPD glycopyronium has similar efficacy and safety to tiotropium, but provides a faster onset of action compared with tiotropium on the first day of therapy.

## Conclusions

Based on the results of the above mentioned studies, glycopyrronium has proven to be capable of inducing favourable effects on lung hyperinflation and its functional and clinical consequences. Bronchodilation afforded by glycopyrronium is more rapid than that of tiotropium since the first dose, and maintains this effect all over 24 hours with a single daily dose. This certainly represents an advantage in terms of adherence to therapy, because it is well known that the efficacy of a therapy also depends on the patient’s adherence to treatment, that must be agreed by the patient once the therapeutical plan has been justified and explained in all details.

Among the crucial factors for adherence, particularly important are the easiness and reliabity of the device and the dosing regimen [46], especially for COPD patients who mostly are old and can present cognitive defects. In fact, incorrect use of inhalation devices is not rare in COPD and it may be determined not only by patient-related factors, but also by the inhaler characteristics and patient’s education [57-59]. The dose of drug delivered from a DPI depends on a correct handling of the device, the internal resistance of the inhaler, and its ability to generate fine particles that can spread till peripheral airways [60]. In this context the Breezhaler® appears a very reliable and user-friendly device: besides having a low intrinsic resistance facilitating high inspiratory flow rates (in excess of 60 L/min) [44], the fine particle fraction (FPF) generated by Breezhaler**®** is 26.8% of the delivered dose versus 9.8% of HandiHaler® (the DPI to deliver tiotropium) [57] such as the former provides greater mean intrathoracic drug deposition (31% vs. 22%) and lower extrathoracic drug deposition (57% vs. 71%) than the latter. In an open-label, multicenter, two-period, 7-day crossover study [57] including 82 patients with moderate-to-severe COPD were assessed the patient’s corrected inhaler use and the patient’s inhaler preference for Breezhaler® and Handihaler® relatively to the various steps in use. The percentage of patients correctly using the inhaler increased from 1^st^ to 7^th^ day and there was no significant difference between the two devices. On the contrary, patients expressed the preference for Breezhaler® in a significantly higher percentage compared to Handihaler® (61% vs. 31% p < 0.01) because of its greater overall comfort, simplicity and confidence in use (confidence that inhalation of drug has been correctly performed).

Semplification of therapeutical regimen by reducing the number of doses to take led to a greater adherence to treatment in patients affected with chronic diseases and mainly with COPD [61,62]. In addition, a lower adherence to treatment has been found to cause a marked worsening of health status [63], whereas the adherence to inhalant therapy in COPD is associated with a lower risk of death and hospitalization for acute exacerbations [46]. Even the rapidity of action of a drug and the perception of the effect it produces when correctly taken according to physician’s instructions are important factors to strengthen the adherence to therapy. In fact, it has been demonstrated that patients more adherent to therapy are those who take it correctly, report a substantial improvement due to therapy, and think their doctor is an effective support [64]. Thus, in relation to these issues glycopyrronium appears particularly reliable due to rapidity of its action, the easiness of inhaler, and the clinically important long-lasting bronchodilation and symptoms control it provides.

As to concerns the characteristic of particularly long bronchodilation afforded by GB, Beeh [45] points out that, differently from short-acting or twice daily bronchodilators, after GB administration there is a marked increase in AUC _0-24_ of FEV_1_ and an increased value of trough FEV_1_ in the morning, that is the worst time of day for COPD symptoms [51] particularly in patients with severe disease [65], suggesting that the drug behaves like an endobronchial pharmacological stent that guarantees a continuous patency of the airways. This can positively affect lung hyperinflation and inspiratory capacity because it is conceivable that the greater and persistent bronchodilation, especially at the level of peripheral airways, determines a more complete pulmonary empting during tidal breathing and improves the respiratory mechanics, with consequent decrease in dyspnea and increase in exercise capacity. Such effect has been assimilated to that induced by surgical reduction of lung volume that is successfully performed in patients with upper lobes emphysema (pharmacological lung volume reduction).

## Competing interests

The author declares that he has no competing interests.
